# Supplementary material for: Activating Hidden Magnons With Anomalous Rotational Symmetry
Source: Adv Sci (Weinh). 2026 Nov 9:e78597. Online ahead of print. doi: 10.1002/advs.78597 (PMC13748910; doi:10.1002/advs.78597)
Supplement: Supplementary file 1 [file ADVS-9999-e78597-s001.pdf]

Supporting Information: Activating Hidden Magnons with Anomalous Rotational Symmetry

Dirk Wulferding,[1] Francesco Gabriele,[2] Wojciech Brzezicki,[3,4] Mario Cuoco,[2] Changyoung Kim,[5] Veronica Granata,[6] Mariateresa Lettieri,[2] Anita Guarino,[2] Antonio Vecchione,[2] Rosalba Fittipaldi,[2] and Filomena Forte[2]

[1] *Department of Physics and Astronomy, Sejong University, Seoul 05006, Republic of Korea*
[2] *CNR-SPIN, c/o Università di Salerno, IT-84084 Fisciano (SA), Italy*
[3] *Institute of Theoretical Physics, Jagiellonian University, ulica S. Łojasiewicza 11, PL-30348 Kraków, Poland*
[4] *International Research Centre MagTop, Institute of Physics, Polish Academy of Sciences, Aleja Lotników 32/46, PL*
[5] *Department of Physics and Astronomy, Seoul National University, Seoul 08826, Republic of Korea*
[6] *Department of Industrial, Electronic and Mechanical Engineering of Roma Tre University, IT-00146 Rome, Rome, Italy*

In this Supporting Information, we provide details on the synthesis of the single crystals and the Raman spectroscopy measurements. Additionally, we present the microscopic derivation of the effective exchange model adopted for Ca_2RuO_4, and of the Loudon–Fleury Raman operator. Moreover, we discuss the symmetry properties of the low-energy magnetic excitations, focusing on their behavior under mirror transformations, and theoretically model the polarization dependence of the Raman intensity.

NOTE 1: EXPERIMENTAL METHODS

Single crystals of $Ca_2Ru_{1-x}Mn_xO_4$ with $x = 0, 0.03, 0.05, 0.10$ were grown using a floating zone furnace and characterised prior to synchrotron beamtime. The challenge of growing large crystals of $Ca_2Ru_{1-x}Mn_xO_4$ is related to the abrupt structural transition occurring close to 350 K. In this framework, we found that cooling slowly through the transition resulted in large plate-shaped samples with the $(00L)$ direction perpendicular to the large face and dimensions in μm. Compositions with increased Mn content tended to be larger but with less regular shape and a larger number of crystalline grains.

The structural properties of the samples were investigated by means of X-ray diffraction (XRD), 2D pole figure analysis, and electron back scattered diffraction. These techniques provided detailed insights into the crystallographic orientation, lattice parameters, and overall crystal quality. In Figure S1 we show the XRD patterns for our $Ca_2(Ru_{1-x}Mn_x)O_4$ single crystals (x = 0, 0.03, 0.05, 0.10) collected at room temperature. The diffraction data across all investigated compositions remain consistent with the pristine orthorhombic *Pbca* space group of Ca_2RuO_4. We observe no anomalous peak splittings or new superlattice reflections that would indicate a global transition to a different space group or a macroscopic structural phase transition. As further support, we note that structural data collected on the same single-crystal batches used for the Raman measurements have already been presented in Ref. [1]. While the XRD data show the expected gradual shifts in Bragg peak positions due to the chemical substitution of Ru with Mn, the macroscopic *Pbca* symmetry is globally preserved, proving that the local spin-orbital symmetry breaking occurs in the absence of a macroscopic structural phase transition. Additionally, EDS analysis confirmed the stoichiometric accuracy and spatially homogeneous elemental distribution of the samples (Figure S2).

NOTE 2: MAGNON PARAMETERS AND THERMAL EVOLUTION OF RAMAN-ACTIVE EXCITATIONS

In Table S1, we report the parameters and energies of the first and second one-magnon peaks, extracted from the B_{1g} Raman spectra for samples with 0%, 3%, 5%, and 10% Mn concentrations.

Figure S3 details the temperature-dependent phonon evolution of an A_g mode around 15 meV that strongly couples to spin degrees of freedom [2]. A discontinuous trend through T_N is evident for phonon frequency, linewidth, and intensity, allowing us to estimate T_N as a function of composition. The extracted values are given in Table S2 and compared to reference values.

In Figure S4 we emphasize the suppression of background intensity in the magnetically ordered phase below the two-magnon gap energy ($\sim$ 60 meV [3]).

Figure S5 highlights the thermal evolution of phonon modes in the B_{1g} channel, with black arrows marking phonons that are absent in the Mn-free compound. The light grey spectra at the top of each panel correspond to the A_g

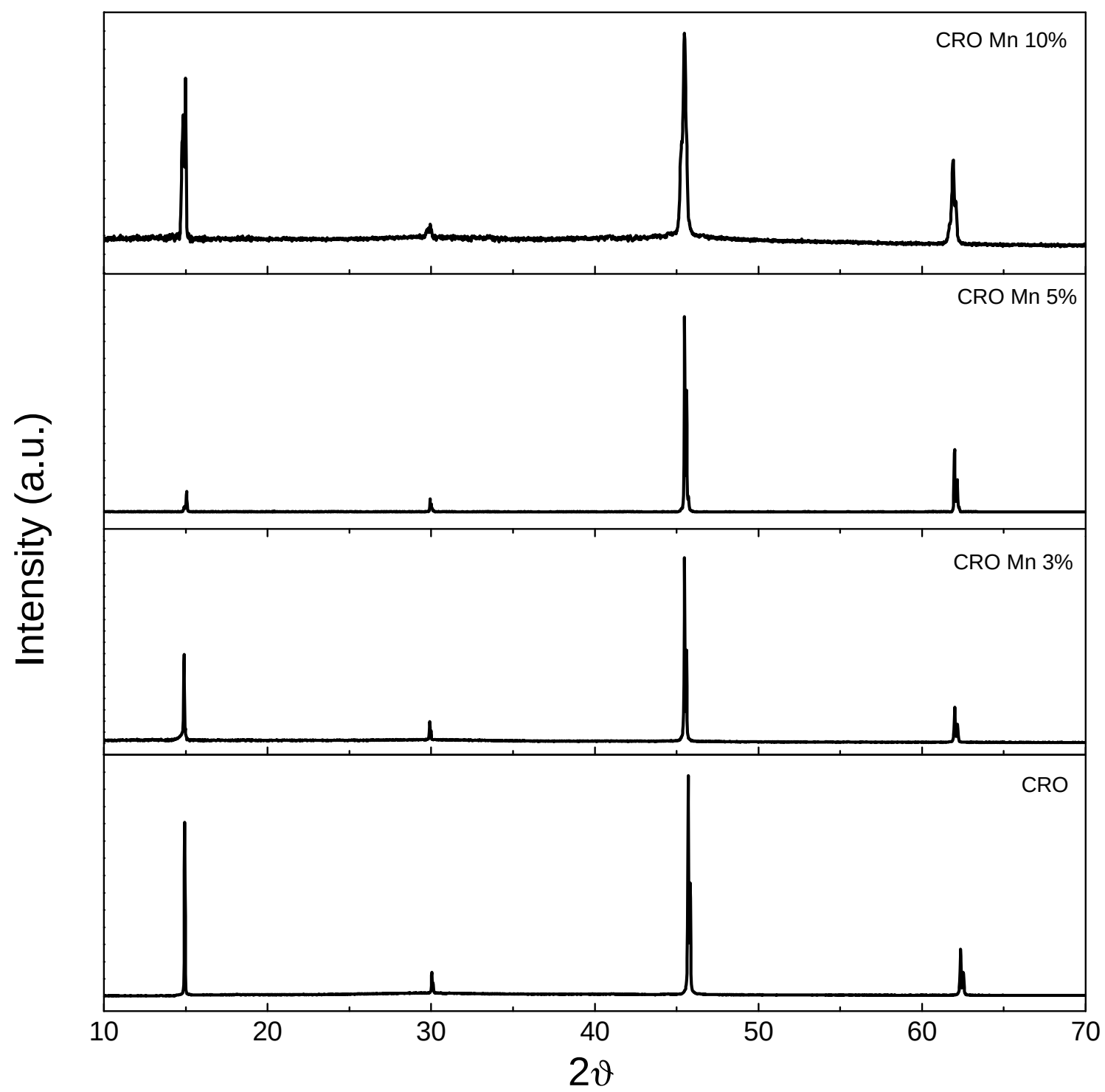

Figure S1. Room-temperature XRD patterns of $Ca_2(Ru_{1-x}Mn_x)O_4$ single crystals for different manganese doping levels (x = 0, 0.03, 0.05, 0.10).

	Energy (meV)	FWHM (meV)	Intensity	Asymmetry
0%	12.59	0.12	0.55	1.5
3%	13.54	0.43	0.29	0.17
	14.69	1.02	0.70	1.55
5%	13.84	0.43	0.23	0.17
	14.84	1.34	0.83	1.25
10%	16.06	1.34	0.84	1.0

Table S1. Composition-dependent parameters: energy, linewidth (FWHM), intensity, and asymmetry (as extracted from the polarization-resolved plots in Figs. 2(c)-(f)) of the one-magnon modes measured at $T = 4$ K.

channel. The 31-meV B_{1g} phonon overlaps energetically with a dominant A_g mode, suggesting a selective A_g phonon activation in the B_{1g} channel through partial Mn-substitution.

Sample	T_N [K] (Ref. [1])	T_N [K] (Raman)
0 %	106.2(7)	110(5)
3 %	116.14(2)	120(5)
5 %	123.1(5)	120(5)
10 %	135.4(4)	140(5)

Table S2. T_N for all four samples taken from a reference study carried out on specimens from the same batch [1] and extracted from temperature-dependent phonon anomalies induced by spin-phonon coupling in the antiferromagnetic phase.

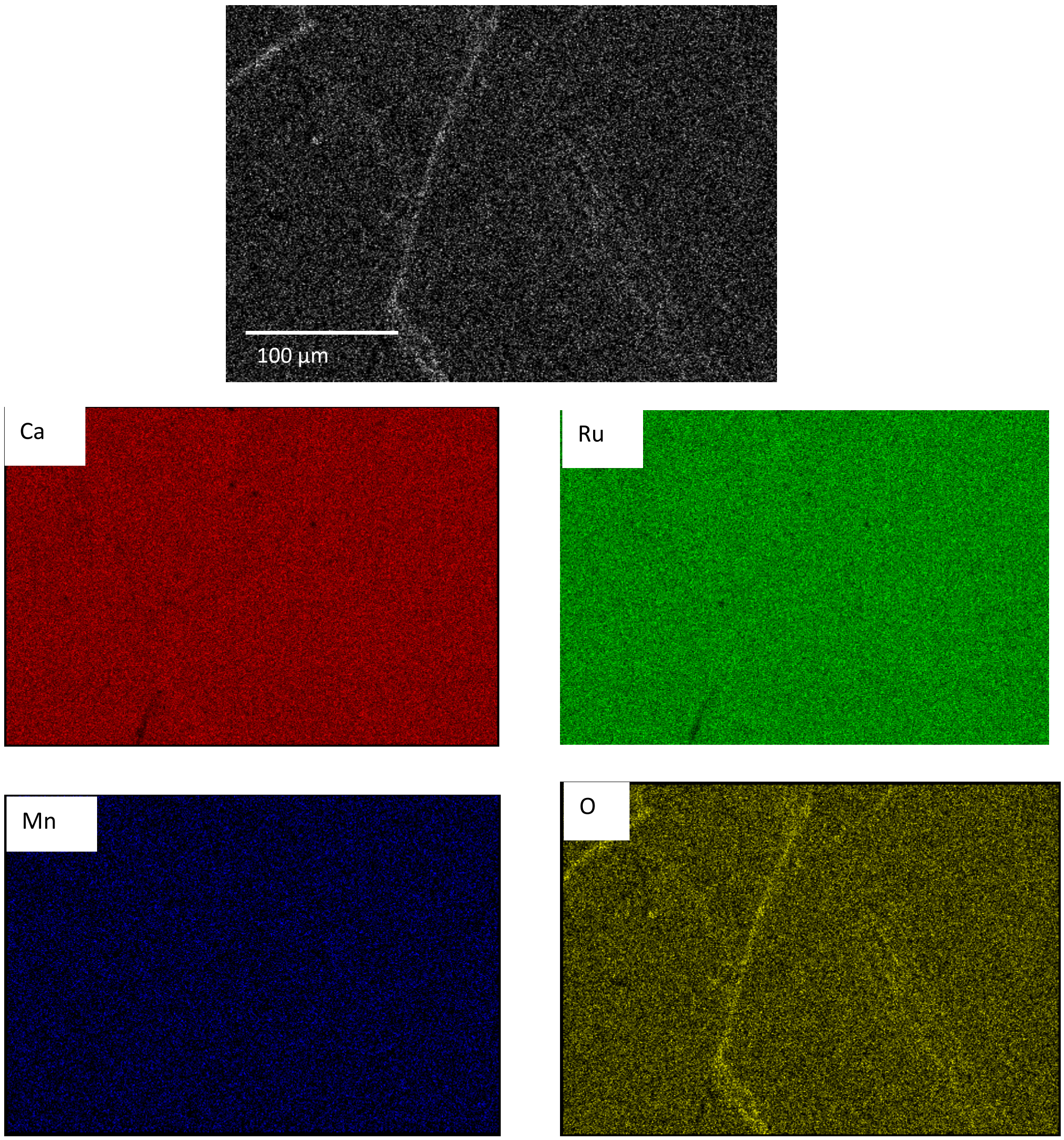

Figure S2. Example of elemental distribution of Ca, Ru, Mn and O in the $Ca_2Ru_{1-x}Mn_xO_4$ with x=0.03.

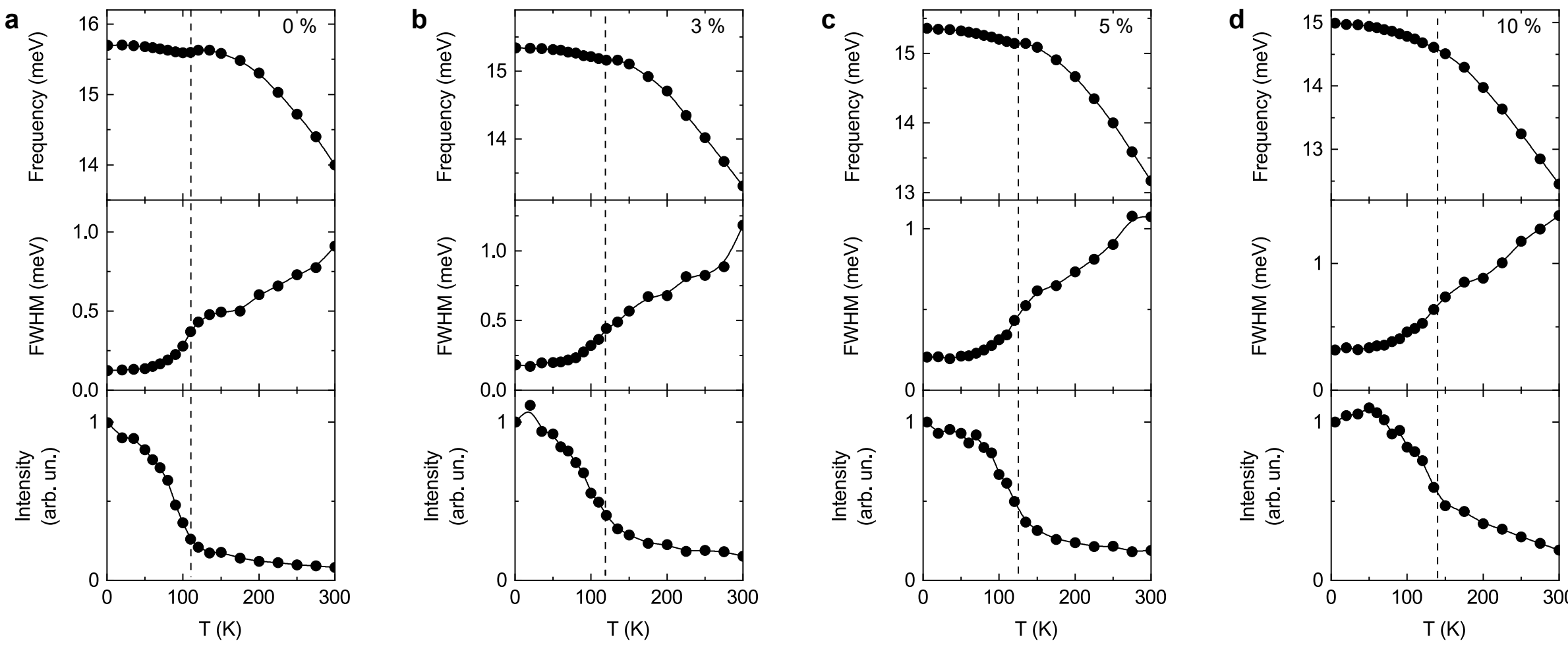

Figure S3. Spin-phonon coupling manifested as phonon anomalies in frequency, linewidth (FWHM) and intensity. Dashed lines mark T_N.

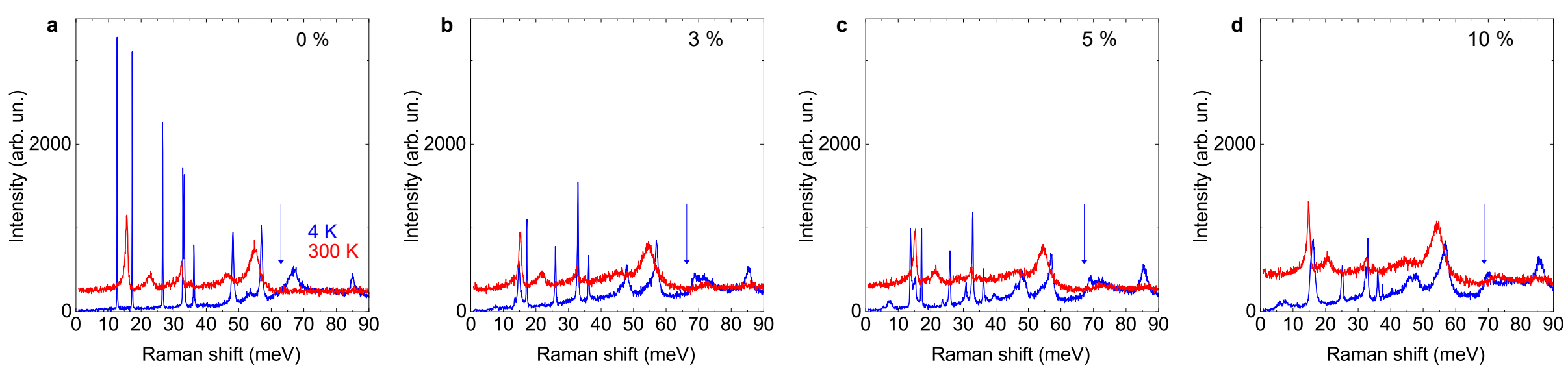

Figure S4. B_{1g} Raman spectra taken in the paramagnetic phase ($T = 300$ K, red curves) vs. spectra taken in the AFM phase ($T = 4$ K, blue curves) for all four compounds. Blue arrows indicate the energy below which spectral weight is suppressed in the magnetically ordered phase.

NOTE 3: DERIVATION OF THE EXCHANGE HAMILTONIAN AND LOUDON-FLEURY RAMAN OPERATOR

In this section, we outline the derivation of the effective spin-1 exchange Hamiltonian and Loudon–Fleury (LF) Raman operator. We start from the local Hamiltonian H_{Ru} describing the t_{2g} electrons on ruthenium ions. Neglecting the rotation and tilting contributions, the rotationally-invariant on-site Coulomb, spin-orbit and crystal field contributions are given by [4–6]

$$\begin{aligned} H_{\text{el-el}}(i) &= U \sum_{\alpha} n_{i\alpha\uparrow} n_{i\alpha\downarrow} - 2J_H \sum_{\alpha<\beta} \mathbf{S}_{i\alpha} \cdot \mathbf{S}_{i\beta} \\ &+ \left(U + \frac{3J_H}{2}\right) \sum_{\alpha<\beta} n_{i\alpha} n_{i\beta} \\ &+ J_H \sum_{\alpha<\beta} d^{\dagger}_{i\alpha\uparrow} d^{\dagger}_{i\alpha\downarrow} d_{i\beta\uparrow} d_{i\beta\downarrow} \end{aligned} \tag{1}$$

$$H_{\text{cf}}(i) = \varepsilon_{xy} n_{i,d_{xy}} + \varepsilon_z \left(n_{i,d_{xz}} + n_{i,d_{yz}}\right), \tag{2}$$

$$H_{\text{SOC}}(i) = \lambda \sum_{\sigma\alpha} \mathbf{L}_{i\sigma} \cdot \mathbf{S}_{i\alpha}, \tag{3}$$

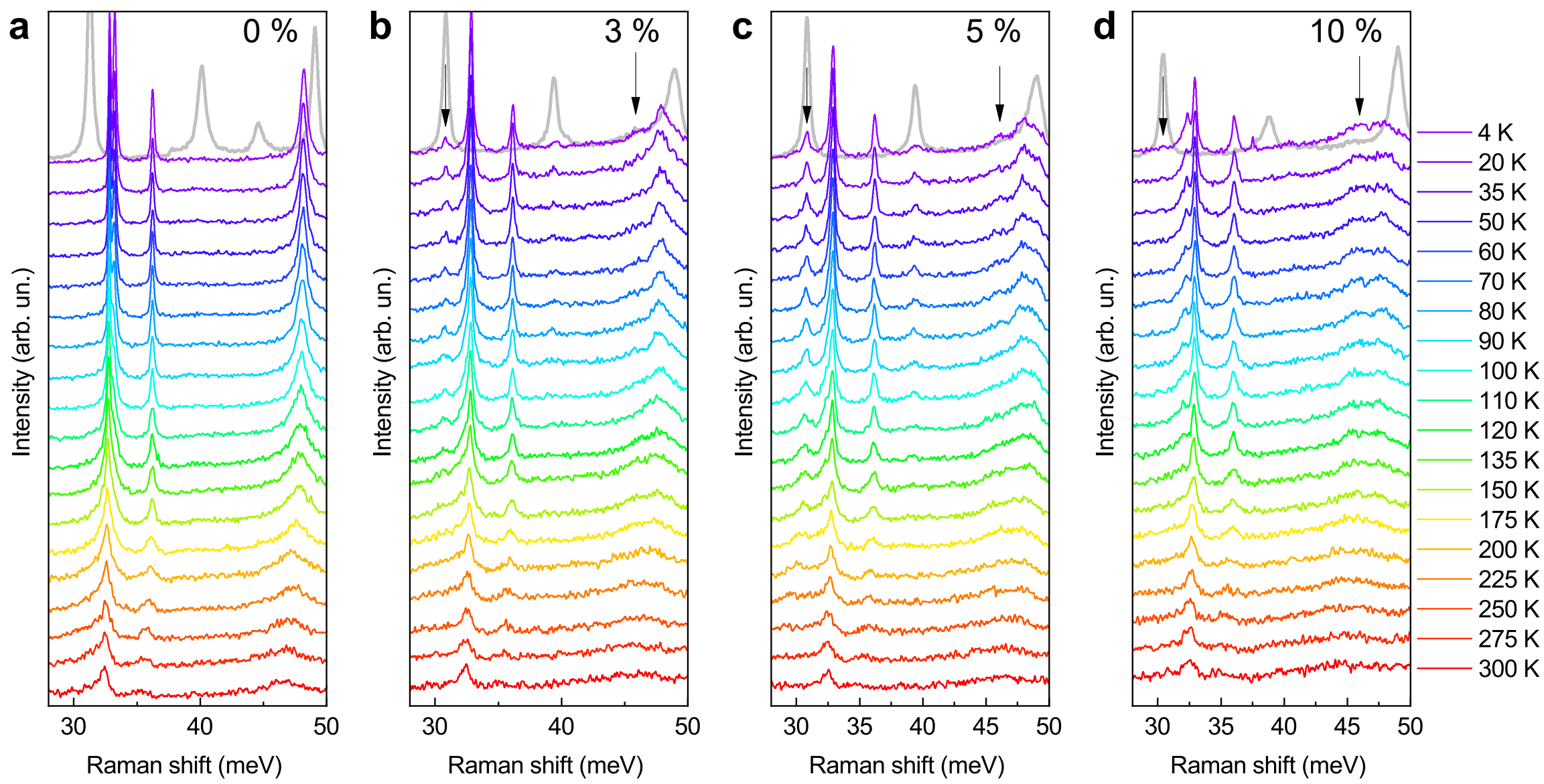

Figure S5. Detailed temperature evolution of phonons in the energy range 28 meV – 50 meV. Black arrows mark phonons observed in the Mn-substituted samples, that are absent in pure Ca_2RuO_4 (0%). Rainbow-colored spectra are obtained in B_{1g} configuration. The light grey lines at the top of each panel are A_g spectra taken at 4 K for comparison.

where i labels the ruthenium site, $\alpha, \beta \in \{d_{xy}, d_{xz}, d_{yz}\}$ label the t_{2g} orbitals, and $d^\dagger_{i\alpha\sigma}$ ($d_{i\alpha\sigma}$) creates (annihilates) an electron of spin σ in orbital α at site i. The corresponding density operator is $n_{i\alpha\sigma} = d^\dagger_{i\alpha\sigma} d_{i\alpha\sigma}$, and $n_{i\alpha} = n_{i\alpha\uparrow} + n_{i\alpha\downarrow}$. The orbital angular momentum and spin operators in second quantization are

$$\mathbf{L}_{j\sigma} = -i \begin{pmatrix} d^\dagger_{j(yz)\sigma} \\ d^\dagger_{j(xz)\sigma} \\ d^\dagger_{j(xy)\sigma} \end{pmatrix} \times \begin{pmatrix} d_{j(yz)\sigma} \\ d_{j(xz)\sigma} \\ d_{j(xy)\sigma} \end{pmatrix}, \qquad \mathbf{S}_{j\alpha} = \frac{1}{2} \begin{pmatrix} d^\dagger_{j\alpha\uparrow} & d^\dagger_{j\alpha\downarrow} \end{pmatrix} \boldsymbol{\tau} \begin{pmatrix} d_{j\alpha\uparrow} \\ d_{j\alpha\downarrow} \end{pmatrix}, \tag{4}$$

where $\boldsymbol{\tau} = (\tau_x, \tau_y, \tau_z)$ denotes the vector of Pauli matrices. In Eqs. (1), (2), and (3), the interaction is parametrized by the intra-orbital Coulomb interaction U, Hund's coupling J_H, the tetragonal energy splitting $\Delta_T = \varepsilon_z - \varepsilon_{xy}$ between the d_{xy} and d_{xz}/d_{yz} orbitals, with energies ε_{xy} and ε_z respectively, and the spin-orbit coupling constant λ. The total local Hamiltonian of the ruthenium ion at site i is therefore

$$H_{\mathrm{Ru}}(i) = H_{\text{el-el}}(i) + H_{\mathrm{cf}}(i) + H_{\mathrm{SOC}}(i). \tag{5}$$

For non-interacting sites, the many-body Hamiltonian is obtained by summing Eq. (5) over i. The coupling between different sites is introduced, at leading order, through the hopping Hamiltonian connecting nearest-neighbor ruthenium ions,

$$H_{\mathrm{Ru-Ru}} = -\sum_{\langle i,j\rangle} \sum_{\alpha\sigma} t^{ij}_\alpha d^\dagger_{i\alpha\sigma} d_{j\alpha\sigma}. \tag{6}$$

The intra-orbital hopping t^{ij}_α depends on the orbital α and bond direction ij, with $ij \parallel x, y$. In the orthorhombic phase, the dominant hopping amplitudes are $t^x_{xy} = t^y_{xy}$ and $t^x_{xz} = t^y_{yz}$, while $t^x_{yz} = t^y_{xz}$ are considerably smaller [7]. For simplicity, we set

$$\begin{aligned} t^x_{xy} &= t, \quad t^x_{xz} = t, \quad t^x_{yz} = 0, \\ t^y_{xy} &= t, \quad t^y_{xz} = 0, \quad t^y_{yz} = t. \end{aligned} \tag{7}$$

In the $t = 0$ limit, the local electron number is conserved. Each ruthenium atom is then in the stoichiometric Ru^{4+} (d^4) configuration, with all four electrons occupying the t_{2g} manifold. For simplicity, we omit the site label i in the

next three equations. As discussed in the main text and in more detail in Ref. [6], the low-energy local manifold relevant to the energy scales probed in the experiment is a quasi-triplet spanned by the following three eigenstates of $H_{\rm Ru}$,

$$
\begin{aligned}
|A_1\rangle &= \frac{1}{\sqrt{1+4a_1^2}}\left(-i\sqrt{2}a_1\,|1,1\rangle - i\sqrt{2}a_1\,|-1,-1\rangle + |0,0\rangle\right), & a_1 &= \frac{\sqrt{2}\lambda}{\Delta_T - \lambda + \sqrt{\Delta_T^2 - 2\Delta_T\lambda + 9\lambda^2}},\\
|A_2\rangle &= \frac{1}{\sqrt{1+2a_2^2}}\left(-i\sqrt{2}a_2\,|-1,0\rangle + |0,1\rangle\right), & a_2 &= \frac{-\Delta_T + \sqrt{\Delta_T^2 + 4\lambda^2}}{2\sqrt{2}\lambda},\\
|A_3\rangle &= \frac{1}{\sqrt{1+2a_3^2}}\left(i\sqrt{2}a_3\,|1,0\rangle + |0,-1\rangle\right), & a_3 &= a_2,
\end{aligned}
\tag{8}
$$

with corresponding energies

$$
E_{A_1} = \frac{\Delta_T - \lambda - \sqrt{\Delta_T^2 - 2\Delta_T\lambda + 9\lambda^2}}{2}, \quad E_{A_2} = E_{A_3} = \frac{\Delta_T - \sqrt{\Delta_T^2 + 4\lambda^2}}{2}. \tag{9}
$$

The states $|l_z, s_z\rangle$ appearing in Eq. (8) describe local d^4 configurations with total orbital angular momentum $L = 1$ and total spin $S = 1$, where $l_z = 1, 0, -1$ and $s_z = 1, 0, -1$ denote the orbital and spin projections along z, respectively. The local basis $\{|A_1\rangle, |A_2\rangle, |A_3\rangle\}$ defines an effective local moment $\mathbf{T}$ with total pseudospin $T = 1$, with

$$
|0\rangle = |A_1\rangle, \qquad |1\rangle = |A_2\rangle, \qquad |-1\rangle = |A_3\rangle, \tag{10}
$$

such that $T^z|m\rangle = m|m\rangle$, for $m = 1, 0, -1$, with corresponding energies $E_0 = E_{A_1}$ and $E_{\pm 1} = E_{A_2}$.

At finite t, the Ru-Ru hopping Hamiltonian does not conserve the local electron number and can therefore connect the local low-energy d^4 manifold to d^3 and d^5 local configurations through nonzero matrix elements. Since $t \ll U$, we treat $H_{\rm Ru-Ru}$ via second-order perturbation theory. Following Refs. [8, 9], the nearest-neighbour exchange Hamiltonian to second order in the hopping amplitude, for a specific bond $i \to j$ chosen along the x axis without loss of generality, is given by

$$
\begin{aligned}
\mathcal{H}_{ij}^x &= \frac{1}{2}\sum_{m,n,r,s}^{\{1,0,-1\}}\sum_{\Gamma,\Gamma'} \langle m|_i \langle n|_j H_{\rm Ru-Ru}|3,\Gamma\rangle_i |5,\Gamma\rangle_j \langle 3,\Gamma|_i \langle 5,\Gamma|_j H_{\rm Ru-Ru}|r\rangle_i |s\rangle_j\\
&\times\left(\frac{1}{E_m + E_n - E_{3,\Gamma} - E_{5,\Gamma}} + \frac{1}{E_m + E_n - E_{3,\Gamma} - E_{5,\Gamma}}\right)|m\rangle_i |n\rangle_j \langle r|_i \langle s|_j\\
&= \frac{1}{2}\sum_{m,n,r,s}^{\{1,0,-1\}}\sum_{\Gamma,\Gamma'}\sum_{\alpha\beta}\sum_{\sigma\sigma'} t_\alpha t_\beta \langle m|_i d_{i\alpha\sigma}^\dagger|3,\Gamma\rangle_i \langle n|_j d_{j\alpha\sigma}|5,\Gamma\rangle_j \langle 3,\Gamma|_i d_{i\beta\sigma'}|r\rangle_i \langle 5,\Gamma|_j d_{j\beta\sigma'}^\dagger|s\rangle_j\\
&\times\left(\frac{1}{E_m + E_n - E_{3,\Gamma} - E_{5,\Gamma'}.} + \frac{1}{E_r + E_s - E_{3,\Gamma} - E_{5,\Gamma'}.}\right)|m\rangle_i |n\rangle_j \langle r|_i \langle s|_j,
\end{aligned}
\tag{11}
$$

where $|3,\Gamma\rangle$ and $|5,\Gamma'\rangle$ denote d^3 and d^5 local configurations with 3 and 5 electrons, respectively, with corresponding local energies $E_{3,\Gamma} = \langle 3,\Gamma| H_{\rm Ru}|3,\Gamma\rangle$ and $E_{5,\Gamma'} = \langle 5,\Gamma'| H_{\rm Ru}|5,\Gamma'\rangle$. Summing over these intermediate configurations yields the effective exchange Hamiltonian

$$
\mathcal{H}_{ij}^x = J_{xx}T_i^x T_j^x + J_{xy}T_i^y T_j^y + J_z T_i^z T_j^z, \tag{12}
$$

where the exchange couplings are given, up to second order in λ, by

$$J_{xx} = \frac{t^2}{U+J_H}\left[1-\left(\frac{5J_H^3 - J_H^2\left(13U+23\Delta_T\right)}{(U+J_H)(U+\Delta_T)(U-3J_H+\Delta_T)}\right.\right.$$
$$\left.\left.+\ \frac{(U+\Delta_T)\left(U^2+12U\Delta_T+6\Delta_T^2\right)J_H\left(17U^2+34U\Delta_T+12\Delta_T^2\right)}{(U+J_H)(U+\Delta_T)(U-3J_H+\Delta_T)}\right)\frac{\lambda^2}{2\Delta_T^2}\right],$$
$$J_{xy} = \frac{t^2}{U+J_H}\left[1-\left(\frac{5J_H^4 + J_H^3\left(8U-2\Delta_T\right)-2J_H\Delta_T\left(U+\Delta_T\right)\left(11U+3\Delta_T\right)}{(U+J_H)(U+\Delta_T)(U-3J_H+\Delta_T)(U+J_H+\Delta_T)}\right.\right.$$
$$\left.\left.+\ \frac{J_H^2\left(2U^2-38U\Delta_T-35\Delta_T^2\right)+\left(U+\Delta_T\right)^2\left(U^2+12U\Delta_T+6\Delta_T^2\right)}{(U+J_H)(U+\Delta_T)(U-3J_H+\Delta_T)(U+J_H+\Delta_T)}\right)\frac{\lambda^2}{2\Delta_T^2}\right],$$
$$J_z = \frac{t^2}{U+J_H}\left[1-\left(\frac{J_H^3+(U+\delta)(U+2\delta)(3U+2\delta)}{(U+J_H)(U+\Delta_T)(U-3J_H+\Delta_T)}\right.\right.$$
$$\left.\left.-\ \frac{J_H(3U+2\delta)(3U+4\delta)+J_H(11U+13\delta)}{(U+J_H)(U+\Delta_T)(U-3J_H+\Delta_T)}\right)\frac{\lambda^2}{2\Delta_T^2}\right]. \tag{13}$$

Summing Eq. (12) over all bonds along x yields the full exchange Hamiltonian $\mathcal{H}_x$, as defined in Eq. (1) of the main text. An analogous expression holds for the exchange Hamiltonian along y, $\mathcal{H}^y$, with $J_{yy} = J_{xx}$ and $J_{yx} = J_{xy}$, as a consequence of Eq. (7). Finally, we note that including the rotation and tilting contributions neglected so far, and treating them at the leading order in perturbation theory, would also yield the full on-site staggered Hamiltonian, as defined in Eq. (2) of the main text.

In a similar fashion, the Raman transition matrix on a single $i \to j$ bond can be computed to second order in perturbation theory in the off-resonant regime—i.e., neglecting the energies of the Raman photons in the perturbative energy denominator—and neglecting their momenta, as[8, 9]

$$R_{ij} = \frac{1}{2}\sum_{m,n,r,s}^{\{1,0,-1\}}\sum_{\Gamma,\Gamma'}\langle m|_i\,\langle n|_j\,\mathbf{j}\cdot\boldsymbol{\epsilon}_{\rm in}\,|3,\Gamma\rangle_i\,|5,\Gamma'\rangle_j\,\langle 3,\Gamma|_i\,\langle 5,\Gamma'|_j\,\mathbf{j}\cdot\boldsymbol{\epsilon}_{\rm out}\,|r\rangle_i\,|s\rangle_j$$
$$\times\left(\frac{1}{E_m+E_n-E_{3,\Gamma}-E_{5,\Gamma'}}+\frac{1}{E_r+E_s-E_{3,\Gamma}-E_{5,\Gamma'}}\right)|m\rangle_i\,|n\rangle_j\,\langle r|_i\,\langle s|_j\,, \tag{14}$$

where the current operator is

$$\mathbf{j} = i\sum_{\langle i,j\rangle}\sum_{\alpha\sigma} t_\alpha^{ij}\mathbf{r}_{ij}d_{i\alpha\sigma}^\dagger d_{i\alpha\sigma}, \tag{15}$$

with $\mathbf{r}_{ij}$ the unit vector along the $i \to j$ bond, and $\boldsymbol{\epsilon}_{\rm in/out}$ the incoming and outgoing polarization vectors of light. Substituting Eq. (15) into Eq. (14), we obtain an expression identical to the perturbative expansion for the energy, up to an overall polarization prefactor $\left(\boldsymbol{\epsilon}_{\rm in}\cdot\mathbf{r}_{ij}\right)\left(\boldsymbol{\epsilon}_{\rm out}\cdot\mathbf{r}_{ij}\right)$. Indeed,

$$R_{ij} = \left(\boldsymbol{\epsilon}_{\rm in}\cdot\mathbf{r}_{ij}\right)\left(\boldsymbol{\epsilon}_{\rm out}\cdot\mathbf{r}_{ij}\right)$$
$$\times\frac{1}{2}\sum_{m,n,r,s}^{\{1,0,-1\}}\sum_{\Gamma,\Gamma'}\sum_{\alpha\beta}\sum_{\sigma\sigma'} t_\alpha t_\beta\,\langle m|_i\,d_{i\alpha\sigma}^\dagger\,|3,\Gamma\rangle_i\,\langle n|_j\,d_{j\alpha\sigma}\,|5,\Gamma'\rangle_j\,\langle 3,\Gamma|_i\,d_{i\beta\sigma'}\,|r\rangle_i\,\langle 5,\Gamma'|_j\,d_{j\beta\sigma'}^\dagger\,|s\rangle_j$$
$$\times\left(\frac{1}{E_m+E_n-E_{3,\Gamma}-E_{5,\Gamma'}}+\frac{1}{E_r+E_s-E_{3,\Gamma}-E_{5,\Gamma'}}\right)|m\rangle_i\,|n\rangle_j\,\langle r|_i\,\langle s|_j$$
$$= \left(\boldsymbol{\epsilon}_{\rm in}\cdot\mathbf{r}_{ij}\right)\left(\boldsymbol{\epsilon}_{\rm out}\cdot\mathbf{r}_{ij}\right)\mathcal{H}_{ij}. \tag{16}$$

Summing Eq. (16) over all bonds yields exactly the LF Raman operator, as defined in Eq. (5) of the main text.

NOTE 4: CONSTRUCTION OF THE GROUND STATES AND LOW ENERGY EXCITATIONS: APPLICATION OF MIRROR SYMMETRY

In this section, we detail the diagonalization of the local Hamiltonian $\mathcal{H}_{loc}$, as defined in Eq. (2) of the main text, and construct approximate single-magnon excited states for the full Hamiltonian $\mathcal{H} = \mathcal{H}_{loc} + \mathcal{H}_x + \mathcal{H}_y$. We further analyze the role of the local mirror symmetry operator $\mathcal{M}_b \equiv \exp\left[i\pi\left(T^x - T^y\right)/\sqrt{2}\right]$, which transforms the local pseudospin components as $\mathcal{M}_b T^{x/y} \mathcal{M}_b = -T^{y/x}$ and $\mathcal{M}_b T^z \mathcal{M}_b = -T^z$, and plays a key role in identifying the Raman-active magnon modes.

The local Hamiltonian, defined on a single site j with either positive or negative signature, reads $\mathcal{H}^j_{\text{loc}} \equiv E_T\left(T^z\right)^2 + (-1)^j\delta_c\left(T^xT^y + T^yT^x\right) + (-1)^j\delta_b\left[T^z\left(T^x+T^y\right) + \left(T^x+T^y\right)T^z\right]$, and can be diagonalized analytically. Since $\mathcal{H}^j_{\text{loc}}$ commutes with $\mathcal{M}_b$, its eigenstates can be further classified according to their parity (positive or negative) under $\mathcal{M}_b$. We first consider the limiting case in which staggered terms are zero, i.e., $\delta_c = \delta_b = 0$. In this case, the Hamiltonian is site-independent, and its three eigenstates can be labeled by the eigenvalues of $\left(T^z\right)^2$ and by their parity under $\mathcal{M}_b$. In terms of the $T=1$ basis states defined in Eq. (10), the lowest-energy state is $|0,-\rangle \equiv |0\rangle$, while the remaining two form a degenerate pair with opposite parity, $|1,\pm\rangle \equiv \left(|-1\rangle \mp i\,|1\rangle\right)/\sqrt{2}$.

In the general case where δ_c and δ_b are nonzero, there exists a unitary transformation $\mathcal{U}_j$ such that $\mathcal{U}^\dagger_j \mathcal{H}^\pm_{\text{loc}} \mathcal{U}_j = \tilde{\mathcal{H}}^j_{\text{loc}}$, where $\tilde{\mathcal{H}}^j_{\text{loc}}$ is diagonal. In particular, we have

$$\tilde{\mathcal{H}}^j_{\text{loc}} = \tilde{E}^j_1 \left|\tilde{1}\right\rangle_j \left\langle\tilde{1}\right|_j + \tilde{E}^j_0 \left|\tilde{0}\right\rangle_j \left\langle\tilde{0}\right|_j + \tilde{E}^j_{-1} \left|-\tilde{1}\right\rangle_j \left\langle-\tilde{1}\right|_j, \tag{17}$$

where $|\tilde{m}\rangle_j$ are eigenvectors, with eigenvalue m, of the spin-1 $z-$components $\tilde{T}^z_j \equiv \mathcal{U}_j T^z \mathcal{U}^\dagger_j$ in the rotated basis. The local energies are

$$\begin{cases} \tilde{E}^j_1 = \frac{E_T + (-1)^{j+1}\delta_c - \sqrt{8\delta_b^2 + \left[E_T + (-1)^{j+1}\delta_c\right]^2}}{2} \\ \tilde{E}^j_0 = E_T - \delta_c - \frac{1+(-1)^j}{4}\left(E_T - \delta_c - \sqrt{8\delta_b^2 + \left(E_T - \delta_c\right)^2}\right) \\ \tilde{E}^j_{-1} = E_T + \delta_c - \frac{1+(-1)^{j+1}}{4}\left(E_T + \delta_c - \sqrt{8\delta_b^2 + \left(E_T + \delta_c\right)^2}\right) \end{cases} \tag{18}$$

with $\tilde{E}^j_1 < \tilde{E}^j_0 < \tilde{E}^j_{-1}$ for all j, and the local eigenvectors can be expressed, in terms of $|0,-\rangle$, $|1,+\rangle$ and $|1,-\rangle$, as

$$\begin{cases} \left|\tilde{1}\right\rangle_j = \frac{1}{\sqrt{1+2\left(\frac{\delta_b}{E^j_1}\right)^2}}\left((1-i)\,\frac{\delta_b}{E^j_1}\,|0,-\rangle + (-1)^{j+1}\,|1,-\rangle\right), \\ \left|\tilde{0}\right\rangle_j = \frac{1+(-1)^j}{2\sqrt{1+2\left(\frac{\delta_b}{E^j_0}\right)^2}}\left((1-i)\,\frac{\delta_b}{E^j_0}\,|0,-\rangle - |1,-\rangle\right) + \frac{1+(-1)^{j+1}}{2}\,|1,+\rangle, \\ \left|-\tilde{1}\right\rangle_j = \frac{1+(-1)^{j+1}}{2\sqrt{1+2\left(\frac{\delta_b}{E^j_{-1}}\right)^2}}\left((1-i)\,\frac{\delta_b}{E^j_{-1}}\,|0,-\rangle + |1,-\rangle\right) + \frac{1+(-1)^j}{2}\,|1,+\rangle. \end{cases} \tag{19}$$

For the parity of the eigenstates, we have

$$\mathcal{M}_b \left|\tilde{1}\right\rangle_j = -\left|\tilde{1}\right\rangle_j, \; \mathcal{M}_b \left|\tilde{0}\right\rangle_j = (-1)^{j+1}\left|\tilde{0}\right\rangle_j, \; \mathcal{M}_b \left|-\tilde{1}\right\rangle_j = (-1)^j\left|-\tilde{1}\right\rangle_j. \tag{20}$$

As already noted in the main text, for each site j, the two excited states have opposite parity with respect to $\mathcal{M}_b$.

The full Hamiltonian $\mathcal{H}$ is no longer invariant under the local mirror $\mathcal{M}_b$, but instead under the global mirror symmetry $\mathbb{M} \equiv M \otimes \prod_j \mathcal{M}^j_b$, where $\mathcal{M}^j_b$ acts locally on site j, and M is the vertical mirror acting on coordinates as $(x,y) \xrightarrow{M} (-y,-x)$. In other words, $[\mathcal{H},\mathbb{M}] = 0$. Since the eigenstates of $\mathcal{H}$ are, in general, non-degenerate, each can be classified according to its parity under $\mathbb{M}$. On the other hand, the Raman operator $\mathcal{R}$ is antisymmetric under $\mathbb{M}$, i.e., $\mathbb{M}\mathcal{R}\mathbb{M} = -\mathcal{R}$, and therefore only connects eigenstates with opposite parity. That is, the Raman cross-section $\langle f|\,\mathcal{R}\,|i\rangle$ is nonzero only if $\mathbb{M}\,|i\rangle = \pm\,|i\rangle$ and $\mathbb{M}\,|f\rangle = \mp\,|f\rangle$.

To fix the ideas, we consider a square lattice with $2N$ sites per side and periodic boundary conditions. The model has, at first approximation, a non-degenerate ground state

$$|\psi_0\rangle \equiv \bigotimes_j \left|\tilde{1}\right\rangle_j , \tag{21}$$

with energy

$$E_0 = 2N^2\left[E_T - \frac{1}{2}\left(\sqrt{8\delta_b^2 + (E_T+\delta_c)^2} + \sqrt{8\delta_b^2 + (E_T-\delta_c)^2}\right)\right], \tag{22}$$

and positive parity under $\mathbb{M}$. Indeed,

$$\mathbb{M}\,|\psi_0\rangle = \bigotimes_j \mathcal{M}_b^j \left|\tilde{1}_{(-1)^j}\right\rangle_j = (-1)^{4N^2}\bigotimes_j \left|\tilde{1}_{(-1)^j}\right\rangle_j = |\psi_0\rangle\,. \tag{23}$$

From $|\psi_0\rangle$ we can build the first excited states by performing a pseudospin flip on a single site. Since the model exhibits translational invariance, the excited states can be sought as single-magnons states, i.e., as superpositions of a spin flip on different sites at zero crystal momentum. Such eigenstates take the general form

$$|\psi\rangle = a\,|\mathbb{0}_+\rangle + b\,|\mathbb{0}_-\rangle + c\,|{-\mathbb{1}_+}\rangle + d\,|{-\mathbb{1}_-}\rangle\,, \tag{24}$$

where the normalized single-magnon vectors on each sublattice are defined as

$$\begin{cases} |\mathbb{0}_+\rangle \equiv \frac{1}{\sqrt{2}N}\sum_{i \text{ even}}\left|\tilde{0}\right\rangle_i \bigotimes_{l\neq i}\left|\tilde{1}\right\rangle_l \\ |\mathbb{0}_-\rangle \equiv \frac{1}{\sqrt{2}N}\sum_{j \text{ odd}}\left|\tilde{0}\right\rangle_j \bigotimes_{l\neq j}\left|\tilde{1}\right\rangle_l \\ |{-\mathbb{1}_+}\rangle \equiv \frac{1}{\sqrt{2}N}\sum_{i,\text{ even}}\left|-\tilde{1}\right\rangle_i \bigotimes_{l\neq i}\left|\tilde{1}\right\rangle_l \\ |{-\mathbb{1}_-}\rangle \equiv \frac{1}{\sqrt{2}N}\sum_{j,\text{ odd}}\left|-\tilde{1}\right\rangle_j \bigotimes_{l\neq j}\left|\tilde{1}\right\rangle_l \end{cases} \tag{25}$$

with a, b, c and d being complex numbers satisfying $|a|^2 + |b|^2 + |c|^2 + |d|^2 = 1$, ensuring that $\langle\psi|\psi\rangle = 1$. Moreover, their parity under $\mathbb{M}$ is

$$\mathbb{M}\,|\mathbb{0}_+\rangle = |\mathbb{0}_+\rangle\,,\quad \mathbb{M}\,|\mathbb{0}_-\rangle = -\,|\mathbb{0}_-\rangle\,,\quad \mathbb{M}\,|{-\mathbb{1}_+}\rangle = -\,|{-\mathbb{1}_+}\rangle\,,\quad \mathbb{M}\,|{-\mathbb{1}_-}\rangle = |{-\mathbb{1}_-}\rangle\,. \tag{26}$$

Each excited state is found by diagonalizing $\mathcal{H}$ within the subspace spanned by $|\mathbb{0}_+\rangle$, $|\mathbb{0}_-\rangle$, $|\mathbb{1}_+\rangle$ and $|\mathbb{1}_-\rangle$. Within this subspace, the diagonal matrix element of the local Hamiltonian are

$$\begin{cases} \langle\mathbb{0}_+|\,\mathcal{H}_{\text{loc}}\,|\mathbb{0}_+\rangle = \left(E_0 + \sqrt{8\delta_b^2 + (E_T-\delta_c)^2}\right), \equiv E_0 + E_{\mathbb{0}_+} \\ \langle\mathbb{0}_-|\,\mathcal{H}_{\text{loc}}\,|\mathbb{0}_-\rangle = \left(E_0 + \frac{E_T - 3\delta_c + \sqrt{8\delta_b^2+(E_T+\delta_c)^2}}{2}\right) \equiv E_0 + E_{\mathbb{0}_-}\,, \\ \langle{-\mathbb{1}_+}|\,\mathcal{H}_{\text{loc}}\,|{-\mathbb{1}_+}\rangle = \left(E_0 + \frac{E_T + 3\delta_c + \sqrt{8\delta_b^2+(E_T-\delta_c)^2}}{2}\right) \equiv E_0 + E_{-\mathbb{1}_+}\,, \\ \langle{-\mathbb{1}_-}|\,\mathcal{H}_{\text{loc}}\,|{-\mathbb{1}_-}\rangle = \left(E_0 + \sqrt{8\delta_b^2 + (E_T+\delta_c)^2}\right) \equiv E_0 + E_{-\mathbb{1}_-}\,, \end{cases} \tag{27}$$

whereas off-diagonal contributions are zero. To compute the matrix elements of the exchange Hamiltonian $\mathcal{H}_x + \mathcal{H}_y$, we note that $\langle\tilde{m}|\,\vec{T}\,|\tilde{m}\rangle = \vec{0}$, so that all diagonal contributions $\langle\mathbb{0}_\pm|\left(\mathcal{H}_x + \mathcal{H}_y\right)|\mathbb{0}_\pm\rangle = \langle{-\mathbb{1}_\pm}|\left(\mathcal{H}_x + \mathcal{H}_y\right)|{-\mathbb{1}_\pm}\rangle = 0$ are zero, and $\langle\mathbb{0}_\pm|\left(\mathcal{H}_x + \mathcal{H}_y\right)|{-\mathbb{1}_\pm}\rangle = \langle{-\mathbb{1}_\pm}|\left(\mathcal{H}_x + \mathcal{H}_y\right)|\mathbb{0}_\pm\rangle = 0$. Then, since

$$\begin{cases} \langle\mathbb{0}_\pm|\left(\mathcal{H}_x + \mathcal{H}_y\right)|\mathbb{0}_\mp\rangle = 0, \\ \langle\mathbb{0}_\pm|\left(\mathcal{H}_x + \mathcal{H}_y\right)|{-\mathbb{1}_\mp}\rangle = -2\left(J_{xx} + J_{xy}\right), \\ \langle{-\mathbb{1}_\pm}|\left(\mathcal{H}_x + \mathcal{H}_y\right)|{-\mathbb{1}_\mp}\rangle = 0, \end{cases} \tag{28}$$

the two lowest-energy single-magnon states $|\psi_-\rangle$ and $|\psi_+\rangle$, with energies $E_- < E_+$ given by

$$E_- = \frac{E_{\mathbb{0}_-} + E_{-\mathbb{1}_+}}{2} - \sqrt{\left(\frac{E_{\mathbb{0}_-} - E_{-\mathbb{1}_+}}{2}\right)^2 + [2(J_{xx} + J_{xy})]^2}, \tag{29}$$

$$E_+ = \frac{E_{\mathbb{0}_+} + E_{-\mathbb{1}_-}}{2} - \sqrt{\left(\frac{E_{\mathbb{0}_+} - E_{-\mathbb{1}_-}}{2}\right)^2 + [2(J_{xx} + J_{xy})]^2}, \tag{30}$$

are

$$|\psi_-\rangle = \frac{1}{\sqrt{1+\alpha_1^2}}\left(|\mathbb{0}_-\rangle + \alpha_1 |{-\mathbb{1}_+}\rangle\right), \quad |\psi_+\rangle = \frac{1}{\sqrt{1+\alpha_2^2}}\left(|\mathbb{0}_+\rangle + \alpha_2 |{-\mathbb{1}_-}\rangle\right), \tag{31}$$

with $\alpha_1 = -\frac{E_- - E_{\mathbb{0}_-}}{2\left(J_{xx}+J_{xy}\right)}$ and $\alpha_2 = -\frac{E_+ - E_{\mathbb{0}_+}}{\left(2J_{xx}+J_{xy}\right)}$. These two eigenstates are labeled according to their parity under $\mathbb{M}$. In particular, since $|\mathbb{0}_-\rangle$, $|\mathbb{1}_+\rangle$ and $|\mathbb{0}_+\rangle$, $|\mathbb{1}_-\rangle$ have opposite parity, the two magnons also exhibit opposite parity. In fact, $\mathbb{M}$ acts on the two excited states as

$$\mathbb{M}\,|\psi_-\rangle = -\,|\psi_-\rangle\,, \quad \mathbb{M}\,|\psi_+\rangle = |\psi_+\rangle\,. \tag{32}$$

We emphasize that, in constructing these approximate eigenstates, we have neglected contributions from multiple spin flips; the exact eigenstates would, in general, include such terms. Nevertheless, their parity would remain unchanged: any additional multiple spin-flip contribution to $|\psi_-\rangle$ would share the parity of $|\mathbb{0}_-\rangle$ and $|{-\mathbb{1}_+}\rangle$, while those contributing to $|\psi_+\rangle$ would match the parity of $|\mathbb{0}_+\rangle$ and $|{-\mathbb{1}_-}\rangle$.

The Raman operator connects the exact excited states $|\Psi_-\rangle$ and $|\Psi_+\rangle$, whose parity is inherited from the approximate $|\psi_-\rangle$ and $|\psi_+\rangle$ above, to the exact ground state $|\Psi_0\rangle$, determining the spectral intensity of the two magnons. Since $|\Psi_0\rangle$ has positive parity, the Raman cross-section is finite for $|\Psi_-\rangle$, which has negative parity, but vanishes for $|\Psi_+\rangle$, which has positive parity. As a result, the first magnon has a finite spectral intensity, whereas the second one is suppressed, as we discuss in greater detail in Note 5.

To conclude, we discuss the role of impurities in activating the second magnon in the Raman response. The presence of local impurities breaks the global mirror symmetry. In this case, even if $\mathbb{M}\mathcal{H}\mathbb{M} \neq \mathcal{H}$, the Hamiltonian can still be decomposed as $\mathcal{H} = \mathcal{H}_{\mathrm{sym}} + \mathcal{H}_{\mathrm{asym}}$, with $\mathcal{H}_{\mathrm{sym/asym}} \equiv \left(\mathcal{H} \pm \mathbb{M}\mathcal{H}\mathbb{M}\right)/2$ such that $\mathbb{M}\mathcal{H}_{\mathrm{sym/asym}}\mathbb{M} = \pm\mathcal{H}_{\mathrm{sym/asym}}$. In particular, $\mathcal{H}_{\mathrm{asym}}$ couples $|\Psi_-\rangle$ and $|\Psi_+\rangle$, yielding a finite matrix element between them. Consequently, the new ground state and two lowest-energy excited states become mixtures of $|\Psi_0\rangle$, $|\Psi_-\rangle$ and $|\Psi_+\rangle$ and no longer possess defined parity. Therefore, the Raman cross-sections for both magnons are finite.

NOTE 5: SINGLE-MAGNON MATRIX ELEMENTS IN THE LOUDON–FLEURY RAMAN OPERATOR

In this section, we explicitly demonstrate why the LF Raman operator $R_{B_{1g}}$, despite being composed of bilinear exchange-like pseudospin operators, yields a non-zero matrix element between the ground state $|\Psi_0\rangle$ and the single-magnon excited states. We show that this process is enabled by local quadrupolar terms induced by octahedral tilts (δ_b), which break spin-flip parity conservation.

The starting point is the Hamiltonian describing the exchange interaction and the local terms arising from the flattening, rotation, and tilting of the RuO_6 octahedra, described by Eqs. (1) and (2) of the main text. Upon defining the ladder operators $T^{\pm} = T^x \pm iT^y$, the spin operators read $T^x = \frac{1}{2}(T^+ + T^-)$ and $T^y = \frac{1}{2i}(T^+ - T^-)$. The quadrupolar term described by the parameter δ_b in $\mathcal{H}_{\mathrm{loc}}$ depends on anti-commutators of the type $\{T^z, T^x\}$ and $\{T^z, T^y\}$. As an example, we consider the first one, which can be rewritten as:

$$\{T^z, T^x\} = \frac{1}{2}\Big(\{T^z, T^+\} + \{T^z, T^-\}\Big). \tag{33}$$

Evaluating the action of $\{T^z, T^+\}$ on the local pseudospin basis:

$$\{T^z, T^+\}\,|{-1}\rangle = \left(T^zT^+ + T^+T^z\right)|{-1}\rangle = T^z\left(\sqrt{2}\,|0\rangle\right) + T^+\left(-\,|{-1}\rangle\right) = -\sqrt{2}\,|0\rangle\,, \tag{34}$$

$$\{T^z, T^+\}\,|0\rangle = \left(T^zT^+ + T^+T^z\right)|0\rangle = T^z\left(\sqrt{2}\,|{+1}\rangle\right) + T^+(0) = \sqrt{2}\,|{+1}\rangle\,, \tag{35}$$

$$\{T^z, T^+\}\,|{+1}\rangle = 0. \tag{36}$$

Similarly, for $\{T^z, T^-\}$:

$$\{T^z, T^-\}\,|{+1}\rangle = \left(T^zT^- + T^-T^z\right)|{+1}\rangle = T^z\left(\sqrt{2}\,|0\rangle\right) + T^-\left(|{+1}\rangle\right) = \sqrt{2}\,|0\rangle\,, \tag{37}$$

$$\{T^z, T^-\}\,|0\rangle = \left(T^zT^- + T^-T^z\right)|0\rangle = T^z\left(\sqrt{2}\,|{-1}\rangle\right) + T^-(0) = -\sqrt{2}\,|{-1}\rangle\,, \tag{38}$$

$$\{T^z, T^-\}\,|{-1}\rangle = 0. \tag{39}$$

These equations demonstrate that the quadrupolar term $\{T^z, T^{x,y}\}$ directly couples states differing by a single spin flip ($\Delta T^z = \pm 1$), specifically connecting $|-1\rangle \leftrightarrow |0\rangle$ and $|0\rangle \leftrightarrow |+1\rangle$. Conversely, it can be shown that the $\mathcal{H}_{\rm loc}$ term, described by the parameter δ_c with $\{T^x, T^y\} = T^x T^y + T^y T^x$, exclusively connects states differing by $\Delta T^z = \pm 2$, mediating direct transitions between $|+1\rangle$ and $|-1\rangle$, and annihilating the state $|0\rangle$. The microscopic implications of these algebraic properties are crucial. We demonstrate this explicitly by evaluating the transition from the many-body ground state to the single-magnon states under the action of the $R_{B_{1g}}$ operator.

In an unperturbed system without octahedral tilts ($\delta_b = 0$), the ground state is generalized with respect to the approximate form considered in Note 3, since it contains virtual two-magnon fluctuations generated by the exchange Hamiltonian, while the excited states are purely single-magnon excitations. Since the exchange-like LF operator $R_{B_{1g}} = \mathcal{H}_x - \mathcal{H}_y$ creates, destroys, or scatters magnons strictly in pairs ($\Delta n = 0, \pm 2$, corresponding to $\Delta T^z = 0, \pm 2$), the zeroth-order matrix element to a single-magnon state identically vanishes.

However, because finite octahedral tilts ($\delta_b \neq 0$) activate local $\Delta T^z = \pm 1$ transitions, spin-flip parity conservation is fundamentally broken. To first order in perturbation theory, the unperturbed ground state acquires a finite one-magnon component, while the single-magnon states $|\psi_\pm\rangle$ acquire corresponding zero- and two-magnon components:

$$|\Psi_0\rangle \approx |\psi_0\rangle + \delta_b \sum_l u_l \, |l\rangle + \dots, \tag{40}$$

$$|\Psi_\pm\rangle \approx |\psi_\pm\rangle + \delta_b \left(v_0^\pm \, |0\rangle + \sum_{\langle i,j \rangle} v_{ij}^\pm \, |i,j\rangle \right) + \dots, \tag{41}$$

where $|0\rangle$ now represents the zero-magnon state, $|l\rangle$ denotes a localized single spin-flip at site l, $|i,j\rangle$ indicates a state with two localized spin-flips at adjacent sites i and j, and u_l, $v_0^\pm$, $v_{ij}^\pm$ are perturbative coefficients. We point out that although these coherent sums over the lattice mathematically constitute delocalized zero-momentum excitations, expanding them in the real-space basis clarifies both the local origin of the δ_b perturbation and the subsequent site-to-site hopping induced by the LF operator.
The Raman transition amplitude $\mathcal{A}_\pm = \langle \Psi_\pm | \, R_{B_{1g}} \, |\Psi_0\rangle$ can then be expanded to linear order in δ_b, yielding two finite scattering channels:

$$\mathcal{A}_\pm \approx \delta_b \, \langle \psi_\pm | \, R_{B_{1g}} \left(\sum_l u_l \, |l\rangle \right) + \delta_b \left((v_0^\pm)^* \, \langle 0| + \sum_{\langle i,j \rangle} (v_{ij}^\pm)^* \, \langle i,j| \right) R_{B_{1g}} \, |\psi_0\rangle \, . \tag{42}$$

To clarify the origin of each term in Eq. (3), we note that the LF operator contains both pair-creation/annihilation terms ($\Delta T^z = \pm 2$, such as $T_i^+ T_j^+$ and $T_i^- T_j^-$) and magnon-conserving exchange terms ($\Delta T^z = 0$, such as $T_i^+ T_j^- + T_i^- T_j^+$). The first matrix element $\langle \psi_\pm | \, R_{B_{1g}} \, |l\rangle$ connects two states that both contain a single magnon. This process is mediated by the $\Delta T^z = 0$ exchange terms, which annihilate the spin flip at site l and recreate it at an adjacent site. In the second channel, $R_{B_{1g}}$ acts via its $\Delta T^z = \pm 2$ pair terms, coupling the zero- and two-magnon fluctuations of the ground state directly to the δ_b-induced zero- and two-magnon components of the excited state. Thus, the local quadrupolar field δ_b acts as the critical mechanism that lifts the strict two-magnon selection rule for the $R_{B_{1g}}$ Raman operator.

However, breaking spin-flip parity conservation is a necessary but not sufficient condition for a finite Raman intensity, as the transition must also satisfy the strict selection rules imposed by the symmetries of the lattice. We therefore evaluate the constraints imposed by the mirror symmetry $\mathbb{M}$. Since $R_{B_{1g}}$ transforms as an odd operator under mirror reflection ($\mathbb{M} R_{B_{1g}} \mathbb{M}^{-1} = -R_{B_{1g}}$) and the ground state is even ($\mathbb{M} \, |\Psi_0\rangle = |\Psi_0\rangle$), inserting the identity operator $\mathbb{I} = \mathbb{M}^{-1} \mathbb{M}$ into the transition amplitude yields:

$$\mathcal{A}_\pm = \langle \Psi_\pm | \, \mathbb{M}^{-1} \mathbb{M} R_{B_{1g}} \mathbb{M}^{-1} \mathbb{M} \, |\Psi_0\rangle = - \langle \Psi_\pm | \, \mathbb{M}^{-1} R_{B_{1g}} \, |\Psi_0\rangle \, . \tag{43}$$

Since $\mathbb{M}$ is a unitary and Hermitian operator, its left action on the excited states yields $\langle \Psi_\pm | \, \mathbb{M}^{-1} = \pm \langle \Psi_\pm|$, depending on their defined parity. We thus arrive at the symmetry constraint:

$$\mathcal{A}_\pm = \mp \mathcal{A}_\pm . \tag{44}$$

This mathematical constraint requires $\mathcal{A}_+ = -\mathcal{A}_+$, immediately implying that the transition to the even magnon state is strictly forbidden ($\mathcal{A}_+ = 0$). In contrast, for the odd magnon state, the relation yields the trivial identity $\mathcal{A}_- = \mathcal{A}_-$, allowing for a finite non-zero transition amplitude.

NOTE 6: MODELING OF POLARIZATION DEPENDENCE OF RAMAN INTENSITY

Far from resonance, the Raman operator $\mathcal{R}$ is symmetric under the exchange of polarization vectors and is described by the Loudon-Fleury term. Close to resonance, the perturbative regime underlying this expression breaks down, and an antisymmetric contribution may emerge from the intermediate-state summation, acquiring significant weight under resonant conditions [8].

In the B_{1g} configuration, defining θ as the angle between the incident polarization vector and e.g. the a crystallographic direction, the Raman operator takes the form:

$$\mathcal{R} \propto \frac{1}{2}\cos(2\theta)R + R_{res} \tag{45}$$

where the first term scales as $\cos(2\theta)$, reflecting a $d_{x^2-y^2}$-wave symmetry of the Raman intensity, while the second term R_{res} is independent of θ. Consequently, under resonant conditions, the total Raman intensity exhibits a constant "background" superimposed on the modulation of the symmetric term.

In Ca_2RuO_4, we assume that R and R_{res} correspond to the Loudon-Fleury and Shastry-like contributions, respectively. Both are odd under the mirror symmetry $\mathbb{M}$ and couple exclusively to the odd magnon state $|\Psi_-\rangle$. The scattering amplitude for the pure system is:

$$A(\theta) = \langle\Psi_-|\mathcal{R}|\Psi_0\rangle = \frac{1}{2}M\cos(2\theta) + M_{res} \tag{46}$$

where $M = \langle\Psi_-|R||\Psi_0\rangle$ and $M_{res} = \langle\Psi_-|R_{res}||\Psi_0\rangle$ are real matrix elements. The resulting intensity is:

$$I(\theta) = |A(\theta)|^2 = \frac{1}{4}M^2\cos^2(2\theta) + M_{res}^2 + MM_{res}\cos(2\theta) \tag{47}$$

The interference term $MM_{res}\cos(2\theta)$ is responsible for the observed angular anisotropy. Based on the preceding equation, we can determine the values of M and M_{res} that best reproduce the Raman intensity ratio between $\theta = 0°$ and 90°, taking the data collected at an incident photon energy of 2.2 eV as a reference (see Figure 2(a) of the main text). These parameters correspond to the observed ratio $I(0°)/I(90°) = I(B_{1g}^{(1)})/I(B_{1g}^{(2)}) = 1.5$ and are obtained for $M_{\mathrm{res}}/M = 0.05$, corresponding to the trend shown in Figure 5g of the main text.

Effect of Impurities: Parity Mixing

In the presence of impurities, states undergo parity mixing. The one-magnon eigenstates $|n_1\rangle$ and $|n_2\rangle$ become orthonormal linear combinations of the even state $|\Psi_-\rangle$ and the odd state $|\Psi_+\rangle$, defined by a mixing angle α:

$$\begin{pmatrix}|n_1\rangle\\|n_2\rangle\end{pmatrix} = \begin{pmatrix}\cos\alpha & \sin\alpha\\ -\sin\alpha & \cos\alpha\end{pmatrix}\begin{pmatrix}|\Psi_-\rangle\\|\Psi_+\rangle\end{pmatrix} \tag{48}$$

To first order, we assume that the macroscopic ground state retains its local spin–orbital ground-state symmetry—namely, it remains even—while the impact of the impurity is strictly confined to the excited states and the local scattering operator. The impurity-modified Raman operator $\mathcal{R}_{\mathrm{imp}}$ now includes terms that couple the ground state to the $|\Psi_+\rangle$ state:

$$\mathcal{R}_{imp} \propto \frac{1}{2}\cos(2\theta)(R^- + R^+) + (R^-_{res} + R^+_{res}) \tag{49}$$

where the operators R^-/R^-_{res} and R^+/R^+_{res} act on the $|\Psi_-\rangle$ and $|\Psi_+\rangle$ states, respectively.
Defining the matrix elements $M^{-,+} = \langle\Psi_{-,+}|R^{-,+}|\Psi_0\rangle$ and $M^{-,+}_{\mathrm{res}} = \langle\Psi_{-,+}|R^{-,+}_{\mathrm{res}}|\Psi_0\rangle$, the transition amplitudes for the two mixed states are:

$$A_1(\theta) = \frac{1}{2}A\cos(2\theta) + B \tag{50}$$

$$A_2(\theta) = \frac{1}{2}C\cos(2\theta) + D \tag{51}$$

where we have defined the effective mixing coefficients:

$$A = M^-\cos\alpha + M^+\sin\alpha, \qquad B = M^-_{res}\cos\alpha + M^+_{res}\sin\alpha \tag{52}$$

$$C = M^+\cos\alpha - M^-\sin\alpha, \qquad D = M^+_{res}\cos\alpha - M^-_{res}\sin\alpha \tag{53}$$

Analysis of the 90° Phase Shift

The observed 90° phase shift between the intensity profiles I_1 and I_2, phenomenologically expressed as $I_2(\theta) = I_1(\theta \pm 90^\circ)$, imposes strict constraints on the scattering amplitudes. Given the trigonometric identity:

$$\cos\big(2(\theta + 90^\circ)\big) = -\cos(2\theta)$$

and assuming that the coefficients A, B, C, and D are real, this relative shift inherently requires the coefficient of the cosine term in I_2 to flip its sign relative to the constant term when compared to I_1. This requirement naturally yields two possible symmetry conditions:

1. $C = -A$ and $D = B$
2. $C = A$ and $D = -B$

Evaluating the first scenario ($C = -A$), we find a direct constraint on the mixing angle α:

$$M^+ \cos\alpha - M^- \sin\alpha = -(M^- \cos\alpha + M^+ \sin\alpha) \implies \tan\alpha = \frac{M^- + M^+}{M^- - M^+} \tag{54}$$

Similarly, the condition $D = B$ yields:

$$\tan\alpha = \frac{M^+_{\text{res}} - M^-_{\text{res}}}{M^+_{\text{res}} + M^-_{\text{res}}} \tag{55}$$

Consequently, a perfect 90° shift between I_1 and I_2 is not a generic feature but a direct consequence of the intrinsic symmetry of the material and the specific values of the resonant and non-resonant matrix elements. Crucially, these conditions can only be satisfied if the mixing angle α is non-zero, making the observed shift a direct manifestation of the mixed-parity states. Indeed, assuming $\alpha = 0$ fundamentally fails to reproduce the experimental observations. In the main text, we report the angular dependence of the intensity for the two one-magnon modes in Figure 5i, for an incident photon energy of 2.2 eV and $x = 5\%$. To model this behavior, we assumed a representative mixing angle $\alpha = \pi/8$. The anisotropic angular dependence of the $|n_1\rangle$ and $|n_2\rangle$ states is optimally reproduced by setting $M^-_{\text{res}}/M^- = 0.08$, in order to get $I(0^\circ)/I(90^\circ) = I(B^{(1)}_{1g})/I(B^{(2)}_{1g}) = 0.15$. For these calculations, the parameters M^+ and M^+_{res} were constrained to precisely satisfy the phase-shift relations in Eqs. (54)–(55). We note that the numerical values of these amplitudes may vary slightly if one aims to optimally reproduce the second peak, as they depend sensitively on the proximity of each mode to its specific resonance condition.

[1] D. G. Porter, F. Forte, V. Granata, M. Cannavacciuolo, R. Fittipaldi, M. Cuoco, A. Bombardi, and A. Vecchione, Guiding antiferromagnetic transitions in Ca_2RuO_4, Scientific Reports **12**, 10957 (2022).

[2] H. Rho, S. L. Cooper, S. Nakatsuji, H. Fukazawa, and Y. Maeno, Lattice dynamics and the electron-phonon interaction in ca_2ruo_4, Phys. Rev. B **71**, 245121 (2005).

[3] S.-M. Souliou, J. Chaloupka, G. Khaliullin, G. Ryu, A. Jain, B. J. Kim, M. Le Tacon, and B. Keimer, Raman Scattering from Higgs Mode Oscillations in the Two-Dimensional Antiferromagnet Ca_2RuO_4, Phys. Rev. Lett. **119**, 067201 (2017).

[4] M. Cuoco, F. Forte, and C. Noce, Interplay of coulomb interactions and c-axis octahedra distortions in single-layer ruthenates, Phys. Rev. B **74**, 195124 (2006).

[5] M. Cuoco, F. Forte, and C. Noce, Probing spin-orbital-lattice correlations in $4d^4$ systems, Phys. Rev. B **73**, 094428 (2006).

[6] L. Das, F. Forte, R. Fittipaldi, C. G. Fatuzzo, V. Granata, O. Ivashko, M. Horio, F. Schindler, M. Dantz, Y. Tseng, D. E. McNally, H. M. Rønnow, W. Wan, N. B. Christensen, J. Pelliciari, P. Olalde-Velasco, N. Kikugawa, T. Neupert, A. Vecchione, T. Schmitt, M. Cuoco, and J. Chang, Spin-Orbital Excitations in Ca_2RuO_4 Revealed by Resonant Inelastic X-Ray Scattering, Phys. Rev. X **8**, 011048 (2018).

[7] W. Brzezicki, F. Forte, C. Noce, M. Cuoco, and A. M. Oleś, Spin-orbital mechanisms for negative thermal expansion in Ca_2RuO_4, Phys. Rev. B **107**, 104403 (2023).

[8] B. S. Shastry and B. I. Shraiman, Theory of Raman scattering in Mott-Hubbard systems, Phys. Rev. Lett. **65**, 1068 (1990).

[9] B. S. Shastry and B. I. Shraiman, Raman scattering in Mott-Hubbard systems, International Journal of Modern Physics B **5**, 365 (1991).
